# Whole-exome sequencing is feasible on a fresh-frozen skin sample of intravascular large B cell lymphoma

**DOI:** 10.1007/s10238-024-01308-0

**Published:** 2024-03-05

**Authors:** Filippo Bagnoli, Giuditta Pini, Bachisio Ziccheddu, Arturo Bonometti, Silvia Alberti-Violetti, Luigia Venegoni, Giuseppe Isimbaldi, Matteo Claudio Da Vià, Angela Ferrari, Luca Baldini, Antonino Neri, Francesco Onida, Niccolò Bolli, Emilio Berti

**Affiliations:** 1 Hematology Unit, Fondazione IRCCS Ca’ Granda Ospedale Maggiore Policlinico, Building Marcora, Via F. Sforza, 35, 20122 Milan, Italy; 2 Department of Oncology and Onco-Hematology, University of Milan, Via Festa del Perdono, 7, 20122 Milan, Italy; 3 Division of Hematology, Department of Medicine, University of Miami Miller School of Medicine, 1600 NW 10 Ave #1140, Miami, FL 33136 USA; 4 Sylvester Comprehensive Cancer Center, University of Miami Miller School of Medicine, 1475 NW 12 Ave, Miami, FL 33136 USA; 5 Pathology Unit, Humanitas Clinical and Research Center IRCCS, Via Alessandro Manzoni, 56, 20089 Rozzano, Italy; 6 Dermatology Unit, Fondazione IRCCS Ca’ Granda Ospedale Maggiore Policlinico, Via Pace, 9, 20122 Milan, Italy; 7 Department of Pathophysiology and Organ Transplantation, University of Milan, Via Festa del Perdono, 7, 20122 Milan, Italy; 8 Pathology Unit, Deparment of Hematology, Oncology, and Molecular Medicine, Niguarda Cancer Center, ASST Grande Ospedale Metropolitano Niguarda, Piazza dell’Ospedale Maggiore, 3, 20162 Milan, Italy; 9 Hematology Unit, Azienda USL-IRCCS di Reggio Emilia, Via Giovanni Amendola, 2, 42122 Reggio Emilia, Italy; 10 Scientific Directorate, Azienda USL-IRCCS di Reggio Emilia, Via Giovanni Amendola, 2, 42122 Reggio Emilia, Italy; 11 Inter-Hospital Division of Pathology, IRCCS MultiMedica, Via Milanese, 300, 20099 Sesto San Giovanni, Italy; 12 Oncoematologia, Ospedale Fatebenefratelli e Oftalmico, Milan, Italy

**Keywords:** Intravascular lymphoma, B-cell lymphoma, Exome, Next-generation sequencing

## Abstract

Intravascular large B-cell lymphoma (IVLBCL) is a rare aggressive extranodal non-Hodgkin lymphoma. The predominant, if not exclusive, growth of neoplastic cells within the lumina of small-sized vessels represents the hallmark of the disease. Diagnosis is challenging due to the absence of marked lymphadenopathy, the highly heterogeneous clinical presentation, and the rarity of the condition. Clinical presentation is characterized by variable combinations of nonspecific signs and symptoms (such as fever and weight loss), organ-specific focal manifestations due to altered perfusion, and hemophagocytic syndrome. The rarity of this entity and the paucity of neoplastic cells in biopsy samples hamper the study of recurrent molecular abnormalities. The purpose of this study was to explore the feasibility of a different approach to recover a sufficient amount of DNA of acceptable quality to perform next-generation sequencing studies. Here, we report the findings of whole-exome next-generation sequencing performed on a fresh-frozen cutaneous sample of IVLBCL, paired with the patient saliva used as germline DNA. To increase the cancer cell fraction, only the subcutaneous tissue was selected. With this approach, we obtained high-quality DNA and were able to identify oncogenic mutations specific for this entity and recapitulating its post-germinal center origin, even if the tumor fraction was low. Molecular studies performed on fresh-frozen cutaneous sample are feasible in IVLBCL, especially when analysis is restricted to the subcutaneous tissue. Wide adoption of this reproducible and cost-effective approach may foster further studies, which may be of help in supporting diagnosis, providing pathogenetic insights, and guiding treatment decisions.

**Supplementary Information:**

The online version contains supplementary material available at 10.1007/s10238-024-01308-0.

## Introduction

Intravascular large B cell lymphoma (IVLBCL) is a rare extranodal B-cell non-Hodgkin lymphoma, accounting for less than 1% of all cutaneous lymphomas with an incidence rate around one case per ten million people per year [1–3]. The hallmark of this entity is the exclusive growth of neoplastic lymphocytes within the lumina of small-sized vessels. This typical growth pattern has some relevant consequences: the multifaceted clinical presentation, which varies depending on the organs affected; the delay in a timely diagnosis due to misleading clinical suspicion; and the difficulty to retrieve a sufficient number of cells for molecular studies. Any organ may be involved, although lymph nodes are usually spared, whereas central nervous system (CNS) and skin are commonly affected. Indeed, in most cases, there are no lymphadenopathies nor tumor masses at diagnosis [2, 4]. Nonspecific systemic symptoms, such as fever, weight loss and night sweats, may be present. Three main clinical variants are described, with different worldwide distribution and diverse treatment outcomes: cutaneous, classical, hemophagocytic syndrome-associated [5].

Histological examination of tissue biopsy with demonstration of tumor cells is essential for diagnosis. Some sites (such as the CNS) require particularly invasive procedures and/or are encumbered by severe complication risk (such as bleeding for kidney). Random skin biopsy has been increasingly shown as the highest yield approach with a great risk–benefit tradeoff [6–8]. In fact, its yield is usually higher than that of bone marrow biopsy, even in those patients without clinically apparent cutaneous involvement [6]. Liquid biopsy approaches have recently gained attention, since concentrations of cell-free DNA are exceptionally high in this entity. Nevertheless, no pathognomonic mutations have been found so far, and therefore this tool cannot be used as the only diagnostic method [7–9].

Given the aggressive behavior of the IVLBCL, deferring diagnosis may lead to multi-organ failure, poor chemotherapy tolerability and fatal complications. Outcomes have been rather improved by increased awareness of the disease (leading to ante-mortem diagnosis) and the addition of the anti-CD20 monoclonal antibody rituximab to CHOP chemotherapy regimen (R-CHOP). Recently, due to the high risk of CNS dissemination at relapse (around 25% at 3 years) [10], the Japanese PRIMEUR-IVL study provided evidence for a new standard of care based on the frontline combination of R-CHOP with a CNS-oriented strategy made of high-dose methotrexate and intrathecal chemotherapy [11]. Nevertheless, around 25–45% [11, 12] patients still relapse, and may die of IVLBCL.

As for nodal subtypes of diffuse large B-cell lymphoma (DLBCL) [13, 14], genomic studies may be of help in the diagnostic workup as well as in prognostic stratification, treatment decisions and follow-up. So far, the low number of neoplastic cells in biopsy specimens and the rarity of this entity have hampered biological studies. To circumvent this problem, in the last years some authors performed targeted next-generation sequencing (NGS) on DNA derived from microdissected tumor cells [7, 15], and the same DNA source has been used for whole-exome sequencing (WES) on post-mortem IVLBCL samples [16], as wells as for WES and RNA-sequencing in the NK/T cell counterpart of this entity [17]. More recently, some groups used cell-free DNA (cfDNA) [7, 9] or mononuclear cells from the bone marrow (BMMNC) [9] to apply WES or smaller targeted gene panels. All the available data point toward an activated B-cell origin of this disease [18], with recurrent mutations in CD79B and MYD88, both occurring in around 50% of cases, frequently in association [7, 9, 15, 16]. However, source material for genomic studies remains a problem: not all cases show bone marrow involvement, cfDNA may be a problematic source of DNA, and tumor cells microdissection is a time-consuming and poorly reproducible technique that may not yield enough DNA for genomic studies.

In this paper, we hypothesized that DNA derived from a fresh-frozen skin biopsy—a routinely used procedure to obtain diagnosis—could be suitable for whole-exome sequencing together with DNA obtained from saliva as germline control.

## Materials and methods

The study involved the use of human samples, which were collected after written informed consent was obtained. All samples were derived from the same single patient. Samples and data were obtained and managed in accordance with the Declaration of Helsinki. The study was approved by the institutional review board of the Fondazione IRCCS Ospedale Maggiore Policlinico of Milan, Italy, and conducted following the institutional guidelines for retrospective studies.

### Sample preparation

The saliva sample was collected using the Oragene DNA OG-500 kit (DNA Genotech Inc., Ottawa, Ontario, Canada) according to manufacturer protocol, and then stored at − 40 °C.

After incisional skin biopsy, the pathologic skin specimen was frozen and stored using liquid nitrogen. In order to maximize neoplastic cell fraction, the fresh-frozen skin sample was further macro-dissected to include the subcutaneous part only. In fact, subcutaneous tissue has low cellularity and at the same time is enriched in small-sized blood vessels. Neoplastic cell cellularity was therefore estimated to increase from 5% to around 30%. A subcutaneous fraction of 2 × 2 × 2 mm was eventually used to extract genomic DNA.

### Genomic DNA (gDNA) purification and quality control

Genomic DNA was isolated from fresh frozen pathologic skin or 100 μL saliva using QIAamp DNA Micro kit (Qiagen, Hilden, Germany) following the manufacturer protocol. Before DNA isolation, the subcutaneous sample underwent tissue digestion using proteinase K as per manufacturer protocol. The gDNA concentration was assessed by Qubit® 2.0 Fluorometer (Invitrogen, Carlsbad, CA, USA), whereas the gDNA degradation status was assessed by DNA Integrity Number (DIN) value on TapeStation 2200 (Agilent Technologies, Santa Clara, CA, USA).

### Whole exome sequencing (WES) library preparation and sequencing

A total of 100–300 ng of each gDNA sample based on Qubit quantification were mechanically fragmented on a E220 focused ultrasonicator Covaris (Covaris, Woburn, MA, USA). Sheared gDNA were used to perform end repair, A-tailing and adapter ligation with Agilent SureSelect XT kit (Agilent Technologies) following the manufacturer instructions. The libraries were captured using Agilent SureSelect Human All Exon v7 (Agilent Technologies) and finally amplified and sequenced on the NovaSeq 6000 platform, 2 × 100 bp (Illumina). The pair end reads were aligned to the reference human genome (GRCh37) using Burrows-Wheeler Aligner (BWA-MEM, v0.7.12). Then, the reads were post-processed using Picard and samtools to remove the duplicated and unmapped reads respectively and Genomic Analysis Toolkit (GATK v4) to left alignment of small insertions and deletions, indel realignment and base quality score recalibration.

## Results

### Patient

**Fig. 1:**
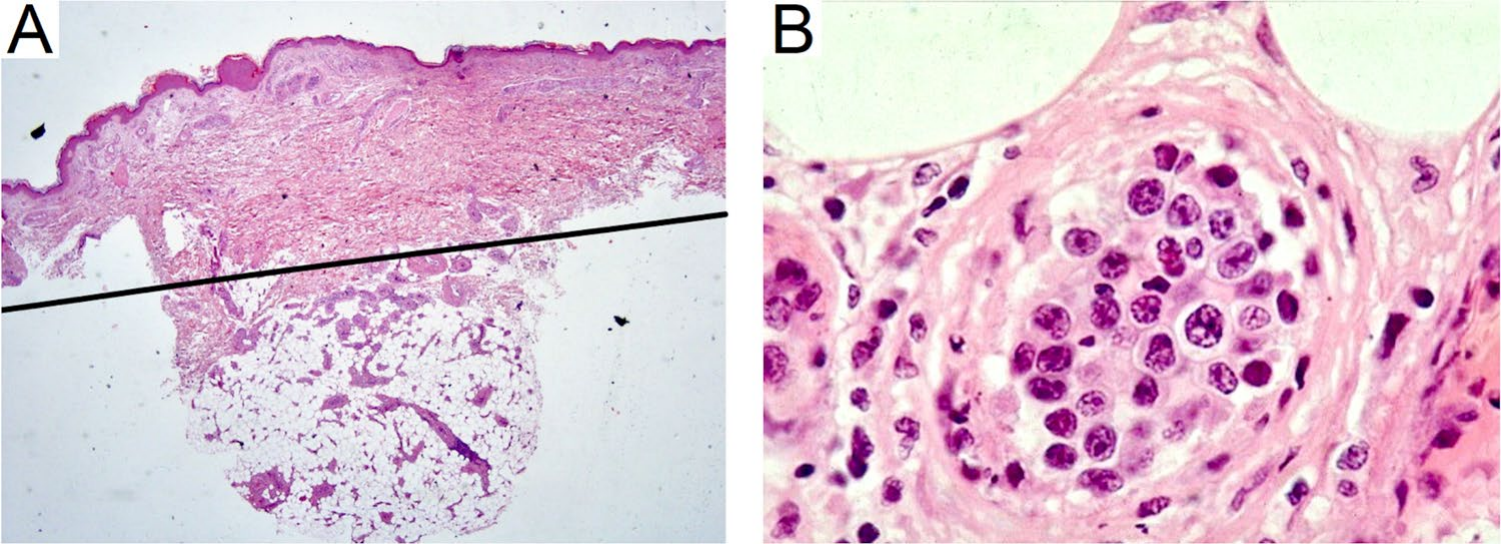
**Panel A** displays low magnification (4 ×) image of hematoxylin and eosin-stained skin tissue from the patient at diagnosis. The typical histological pattern is shown, with small vessels in the subcutaneous and dermal layers enlarged and filled with neoplastic cells, which spare the remaining areas of the skin. The black line represents the approximate cut section performed to include subcutaneous tissue only. **Panel B** shows high magnification (60 ×) of hematoxylin and eosin-stained small vessel in the subcutaneous tissue, filled with packed medium-to-large sized cells, with highly atypical nuclei. The surrounding connective tissue is spared

The clinical history of the patient was previously reported [19]. Briefly, an 84-year-old Italian woman presented to the dermatology clinic with plaque and nodular lesions at both thighs, B symptoms, anemia, and increased lactate dehydrogenase. Therefore, she underwent incisional skin biopsy of one cutaneous lesion, which was divided in two parts, one formalin-fixed for histological analysis and the other part fresh-frozen and finally used for the WES analysis here presented. A saliva sample was stocked too. Histological analysis revealed IVLBCL (Fig. 1). The PET scan revealed multiple areas of increased uptakes on the thighs, at both cutaneous and subcutaneous sites. Bone marrow biopsy was negative, as well as MRI scan of central nervous system. The patient received R-COMP treatment regimen which led to complete remission, but relapsed 5 months later. Low-dose oral cyclophosphamide and dexamethasone were then started for disease control with good efficacy. The patient died one year later due to acute myocardial infarction.

### Genomic DNA quality and sequencing metrics

From the tumor sample we obtained 864 ng of DNA of good quality, with a DNA integrity number (DIN) of 9 suggesting that small fresh frozen skin biopsies can be easily used for NGS analyses. Germline DNA was obtained from saliva with a DIN of 8.

Quality control of the sequencing was also satisfactory: for the tumor sample, the on-target mapping and PCR duplicates accounted for 79.44 and 18.25%, respectively. Figures were similar for the germline saliva sample, with 79.19% on-target reads and 18.81% PCR duplicates. The on-target depth was 90 × for the germline sample and 173 × for the tumor specimen.

### Detection of mutations

To increase stringency of analysis in our sample, where low purity was expected and artifacts were possible, we employed three mutational callers (MuTect2, Seurat, and Strelka) and filters for known germline variants in addition to simultaneous analysis of the matched germline sample. We only selected variants called by at least 2 callers. To remove false positives and polymorphisms we excluded all the variants that either: had a tumor log of odds score (TLOD score) lower than 6.5 from MuTect2; were not listed in the Catalogue Of Somatic Mutations In Cancer (COSMIC) database; were included in polymorphism databases (gnomAD, NCBI dbSNP) without a COSMIC annotation, and were therefore considered to be likely germline variants. Only coding variants with potential pathological impact were considered (missense, nonsense, nonstop, splice site). To identify copy number alterations (CNAs) from WES data we applied Excavator2 v1.1 tool as already described [20].

Eventually, 170 of all variants were present in COSMIC and represented faithful cancer variants (see the gene list provided in Supplementary table), which is in line with the number of mutations detected on BMMNC and post-mortem microdissected IVLBCL cells by the other WES studies.

**Fig. 2:**
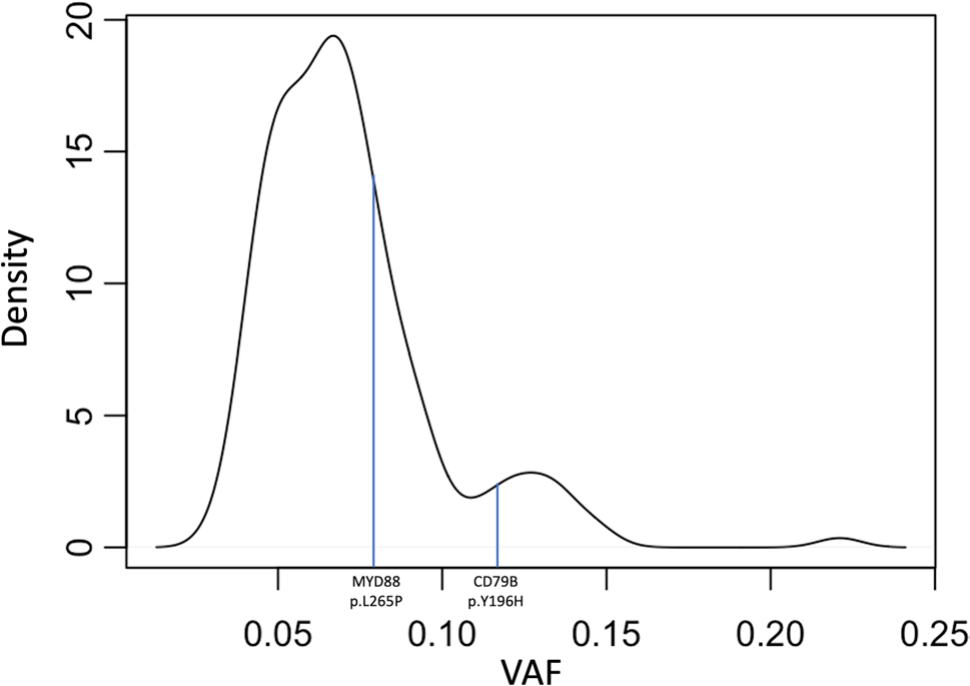
Density plot of allelic frequency distribution for the 170 variants considered after the application of the cited filters. Allelic frequency is represented on the *x*-axis. On the *y*-axis the density is shown as the number of mutations showing a given allelic frequency. The two MYD88 p.L65P and CD79B p.196H variants are shown in the plot, at their variant allele frequency, 0.079 and 0.117, respectively

The median allelic frequency of the final 170 tumor variants was 0.067 (range 5°–95° percentile 0.04–0.12). The distribution of allelic frequency was then analyzed by density plot (Fig. 2). Based on this, we could confirm the removal of germline polymorphisms. Furthermore, the enrichment of mutations with low variant allele frequency (VAF) suggests -as expected- the presence of a high percentage of contaminating normal cells. Based on the distribution of the faithful cancer variants, the tumor fraction of the sample was estimated to represent about 15–30% of the cells sequenced, matching our microscopic estimate. Thus, all the values of allelic frequency should be interpreted considering the elevated amount of contaminating normal cells.

In this one sample report, we first sought to identify recurrent variants that may confirm the robustness of our approach. We identified a CD79B Y196H missense variant with a VAF of 11.7%, which was previously reported as recurrent hotspot variant in IVLBCL [7, 9, 15, 16]. Furthermore, we identified a canonical MYD88 L265P mutation at a VAF of 7.9%, also described as recurrent in this disease [7, 9, 15, 16].

Genes involved in epigenetic regulations were recognized as well. The KMT2D gene has been described as mutated in IVLBCL [9]; our sample carries a different mutation, the E1658* nonsense variant with a VAF of 9.4%, which has been described in other cancers though [21]. Furthermore, the disruption of epigenetic regulators has recently been shown to be a recurrent feature in IVLBCL, as well as in DLBCL and intravascular NK-/T-cell lymphoma (IVNKTCL) [9, 13, 14, 17]. The TBL1XR1 gene is frequently affected by mutations in ABC-DLBCL [14, 22], and has been described in IVLBCL [9, 16], as well as in MYD88-wild type lymphoplasmacytic lymphoma cases [23]. Our sample carries the p.V228D mutation with a VAF of 6.5%, which occurs in a different codon and has been described in nodal marginal zone lymphoma [24].

Notably, no mutations occurring in potential players of the intravascular distribution (such as CD29 a CD54) have been found. Among other variants, we could find a CD44 P16S missense variant, with a VAF of 5.5%, which was previously reported in relapsed/refractory DLBCL [25], but not in IVLBCL case series by Shimada and collaborators, who found a CD44 mutation in a different codon [9]. Targeted approaches did not test the CD44 gene. Moreover, also a CD9 missense variant with a 14.5% VAF was found in our sample, which is another gene that may play a role in intravascular localization of the disease.

Finally, we could confirm post-germinal center origin in our sample by identifying mutations in genes known to be targeted by off-target activation-induced cytidine deaminase (AID) activity -a process known as aberrant somatic hypermutation (SHM)-, such as PIM1, TMSB4X, MPEG1 and OSBPL10. No significant CNAs were identified.

## Discussion

IVLBCL is a rare entity with poor representation of neoplastic cells in biopsy specimens. Histological analysis of pathological samples is mandatory for diagnosis. Several recent studies showed that random skin biopsy is the approach with the highest diagnostic yield and the lowest side effect and complication rate [6–8, 26]. Molecular studies are needed to better understand pathogenesis of this disease, to evaluate the prognostic impact of specific genomic alterations, to guide treatment decisions and new treatment development, and to accurately detect early relapse during follow-up.

In consideration of all these elements, we investigated whether skin samples collected during the diagnostic work-up could be used for whole-exome next generation sequencing. Several limitations had to be addressed to get appropriate material for comprehensive genomic studies. First, DNA quality is of paramount importance for subsequent analyses. To this issue, we used fresh-frozen material which is known to allow excellent tissue preservation and to avoid artifacts arising from formalin-fixation. Furthermore, high quality DNA allows to work with limiting DNA quantity. Second, the neoplastic cell fraction is extremely low in tissue biopsies, since tumor cells are located exclusively in small vessels which can’t be extensively and selectively sampled. To solve this problem, we were able to dissect subcutaneous tissue which is enriched in small vessels, and has otherwise low cellularity. This could be performed manually with a scalpel, without the need for laser capture microdissection equipment.

So far, most molecular studies have been limited to targeted-sequencing on DNA derived from either micro-dissected tumor cells or cfDNA [7, 15]. Tumor cell microdissection is an extremely time-consuming and operator-dependent procedure. On the other hand, cfDNA can be problematic too. For example, since no pathognomonic mutations have been found so far, the chance of nonspecific finding is to be considered. As such, MYD88 *L265P* mutation may be the fortuitous finding of other lymphoproliferative disorders -such as IgM monoclonal gammopathy of unknown significance (MGUS), lymphoplasmocytic lymphoma/Waldenstrom macroglobulinemia, or DLBCL-, rather than the rare IVLCBL. Similarly, CD79B *Y196* may be found frequently (around 30%) in activated-B cell (ABC)-DLBCL, especially in those aggressive cases with extranodal clinical presentation, the so called “MCD” or “cluster 5” subgroups, as defined by Schmitz and Chapuy, respectively [13, 14]. Conversely, in our approach, these mutations can be directly assigned to the tumor clone present in the biopsy.

Targeted studies focused on a panel of genes, selected on the assumption of a non-GCB origin of IVLBCL, have been important in confirming aberrations in pathways such as the B-cell receptor (BCR)-signaling pathway, the Toll-like receptor/Interleukin-1 receptor (TLR/IL-1R) pathway, and the nuclear factor kappa B (NFκB) pathway. However, more appropriate and specific gene panels could be established only after comprehensive genome-wide studies. In fact, to date there are no genomic data able to explain the typical histological pattern of this disease nor to confirm mutations in genes whose products were previously described as potential players of intravascular distribution (such as CD29 or CD54) [5].

Until recently, the only attempt of comprehensive genomic studies was performed on DNA derived from both cfDNA and BMMNC, although the cohort was entirely formed of Japanese patients, and therefore may not be representative of the worldwide IVLBCL cohort. Yet no genomic features leading to the typical intravascular pattern have been found. Furthermore, analysis of bone marrow samples is limited to the cases with bone marrow involvement. More recently, a WES study was performed by Kodgule and colleagues on post-mortem tissues involved by IVLBCL [16]. In their study, they found recurrent mutations in the RAC2 gene, and postulated that this mutation may play a role in IVLBCL cells adhesive features. However, in the aforementioned WES study by Shimada and colleagues, RAC2 mutations were identified in only a minority of cases, and this particular finding was not emphasized [9]. We were not able to detect any RAC2 mutations despite thorough evaluation. Given that our analysis was confined to a single patient, and the two other studies reported RAC2 mutations in only a subset of patients, this finding underlines the possibility that, for some IVLBCL cases, exclusive intravascular location may be attributed to different mediators [9]. Further comprehensive studies are needed to evaluate the recurrence and significance of RAC2 mutations in IVLBCL.

In this study, we found that fresh-frozen subcutaneous sample allows comprehensive genomic studies. In fact, DNA quality and quantity were high, and the cancer cell fraction could be artificially enhanced by selection of the subcutaneous selection. The resulting tumor DNA fraction, albeit low, was amenable to sequencing analysis at medium depth. However, it was likely still too low to reliably identify CNAs. Confirming the feasibility and robustness of our approach, our results align with those obtained from workflows using different sequencing techniques, sample types and manipulations, and geographical areas. In fact, we confirmed the two most frequently described mutations in IVLBCL, CD79B and MYD88 [7, 9, 15, 16]. Moreover, we found a similar number of mutations as compared to the two other WES analyses [9, 16]. In line with other reports, we confirmed that our sample bears multiple mutations in genes targeted by SHM (PIM1, TMSB4X, MPEG1 and OSBPL10) [9, 16]. Since we could only analyze one sample, we cannot exclude that genes with multiple mutations in our dataset could also be artefactual or arise from other localized hypermutational processes such as those mediated by aberrant activity of apolipoprotein B mRNA editing enzyme catalytic polypeptide (APOBEC) [27]. Although limited by the small sample size which is partly due to the extreme rarity of this entity, we believe our study may represent an important proof-of-concept which could be further explored. Our methodology, which is time-efficient and cheap, can be adopted by any pathology laboratory, and could be easily integrated in the standard diagnostic workup of the patient, which is based on random skin biopsy even in the absence of skin lesions. The main limitations of our approach include its operator-dependent nature during the cutting of the biopsy specimen and the necessity to freeze the sample which can be resource-intensive. On the other hand, identifying subcutaneous tissue before cutting is easily feasible, and freezing IVLBCL biopsies should not strain resources, given its rarity.

Therefore, this approach, if validated, may be employed in future studies to better define the genomic landscape of this rare entity, through whole genome sequencing studies which can be carried out at increasing depths using modern technologies and can detect subclonal populations [28]. Extensive and widespread adoption of such pipeline may lead to recognize the specific aberrations associated with this entity, its different clinical presentations, response to treatment, and CNS relapse risk. In case pathognomonic mutations should be found, they could also be used as an important tool to confirm diagnosis and guide measurable residual disease assessments in follow-up. Unravelling the characteristic pathogenetic features may in turn lead to personalized treatments through the use of targeted panels [29–31]. Interestingly, MYD88 L265P has already been described as a target of ibrutinib, whereas little is known about CD79 mutations and polatuzumab vedotin efficacy. Moreover, since the microenvironment contribution on disease pathogenesis is likely limited, IVLBCL may be a good model to identify tumor-intrinsic key drivers, as opposed to DLBCL which is much more influenced by interaction with non-neoplastic cells most times.

## Conclusion

In conclusion, fresh freezing a portion of the random skin biopsies performed during the diagnostic work-up and restricting analysis to subcutaneous tissue results is an easily reproducible and cost-effective approach that may be useful for subsequent comprehensive genomic analysis which are still strongly needed in this entity. To the best of our knowledge, this is the first study to use fresh frozen tissue IVLBCL sample collected from living patient for whole-exome sequencing.

## Supplementary Information

Below is the link to the electronic supplementary material.

Supplementary file1 Gene list of mutated genes considered after application of filters. The list shows the mutated genes considered after the application of the filters cited in the main text. The several columns provide different types of info, whereas each line is referred to a single gene. From the left, each column shows: the Hugo Symbol of each gene, the chromosome where the gene is placed, the nucleotide position of the variant of interest (start position, end position), the reference DNA base, the variant DNA base detected, the type of variant identified according to classification variant, the cDNA change, the aminoacidic change, the variant allele frequency, the estimated variant allele frequency corrected by the tumor cell fraction of 15%, the estimated variant allele frequency corrected by the tumor cell fraction of 30%, the number of times in which the reference DNA base has been found in the pathological sample, the number of times in which the variant DNA base has been found in the pathological sample, the number of times in which the reference DNA base has been found in the normal sample, the number of times in which the variant DNA base has been found in the normal sample. (XLSX 29 KB)
